# Mapping Phosphodiesterase 4D (PDE4D) in Macaque Dorsolateral Prefrontal Cortex: Postsynaptic Compartmentalization in Layer III Pyramidal Cell Circuits

**DOI:** 10.3389/fnana.2020.578483

**Published:** 2020-11-20

**Authors:** Dibyadeep Datta, John F. Enwright, Dominique Arion, Constantinos D. Paspalas, Yury M. Morozov, David A. Lewis, Amy F. T. Arnsten

**Affiliations:** ^1^Department of Neuroscience, Yale School of Medicine, Yale University, New Haven, CT, United States; ^2^Department of Psychiatry, Translational Neuroscience Program, School of Medicine, University of Pittsburgh, Pittsburgh, PA, United States

**Keywords:** prefrontal cortex, PDE4D, cAMP, pyramidal cell, calcium, microdomains

## Abstract

cAMP signaling has powerful, negative effects on cognitive functions of the primate dorsolateral prefrontal cortex (dlPFC), opening potassium channels to reduce firing and impair working memory, and increasing tau phosphorylation in aging neurons. This contrasts with cAMP actions in classic circuits, where it enhances plasticity and transmitter release. PDE4 isozymes regulate cAMP actions, and thus have been a focus of research and drug discovery. Previous work has focused on the localization of PDE4A and PDE4B in dlPFC, but PDE4D is also of great interest, as it is the predominant PDE4 isoform in primate association cortex, and PDE4D expression decreases with aging in human dlPFC. Here we used laser-capture microdissection transcriptomics and found that PDE4D message is enriched in pyramidal cells compared to GABAergic PV-interneurons in layer III of the human dlPFC. A parallel study in rhesus macaques using high-spatial resolution immunoelectron microscopy revealed the ultrastructural locations of PDE4D in primate dlPFC with clarity not possible in human post-mortem tissue. PDE4D was especially prominent in dendrites associated with microtubules, mitochondria, and likely smooth endoplasmic reticulum (SER). There was substantial postsynaptic labeling in dendritic spines, associated with the SER spine-apparatus near glutamatergic-like axospinous synapses, but sparse labeling in axon terminals. We also observed dense PDE4D labeling perisynaptically in astroglial leaflets ensheathing glutamatergic connections. These data suggest that PDE4D is strategically positioned to regulate cAMP signaling in dlPFC glutamatergic synapses and circuits, especially in postsynaptic compartments where it is localized to influence cAMP actions on intracellular trafficking, mitochondrial physiology, and internal calcium release.

## Introduction

Many cognitive disorders involve dysfunction of the newly evolved dorsolateral prefrontal cortex (dlPFC) (Arnsten, 2015). The dlPFC subserves our highest cognitive abilities, generating the mental representations that are the foundation of abstract thought and the basis for flexible, goal-directed behavior (Goldman et al., 1971; Fuster, 2001; Robbins and Arnsten, 2009; Datta and Arnsten, 2018). Studies in non-human primate dlPFC have revealed that pyramidal cell microcircuits that mediate visual spatial working memory reside in deep layer III (Goldman-Rakic, 1995). Specifically, persistent firing during working memory arises from the recurrent excitation of pyramidal cells with similar spatial tuning (Goldman-Rakic, 1995; Kritzer and Goldman-Rakic, 1995; Melchitzky et al., 1998; Gonzalez-Burgos et al., 2000). These dlPFC layer III circuits have expanded greatly in mammalian evolution, with elaboration of basal dendritic arbors and density of dendritic spines, allowing amplification of neural connections required for high-order cognition (Elston, 2000, 2003; Elston et al., 2006; Defelipe, 2011; Amatrudo et al., 2012; Medalla and Luebke, 2015; Gilman et al., 2017; Hsu et al., 2017).

Various lines of evidence suggest that the highly ramified dlPFC layer III circuits in primates are uniquely regulated at the molecular level by cyclic AMP-protein kinase A (cAMP-PKA) signaling pathways which can magnify calcium signaling near glutamate synapses and open potassium channels to dynamically gate network connections (Arnsten et al., 2012; Arnsten, 2015). Calcium homeostasis and cAMP levels are determined, in part, by the phosphodiesterases (PDEs), which play a crucial role in regulating cAMP and cGMP signaling pathways (Houslay and Adams, 2003; Baillie et al., 2019). PDEs modulate a plethora of physiological processes, and their dysregulation has been implicated in several brain disorders, including schizophrenia, Huntington’s disease and Alzheimer’s disease (Baillie et al., 2019). Given their relevance for regulating intracellular signaling pathways, PDEs have emerged as novel therapeutic targets for ameliorating cognitive deficits (Baillie et al., 2019).

The cAMP-specific PDE4 isozymes PDE4A, PDE4B, and PDE4D are highly expressed in brain (Baillie et al., 2019), with the PDE4D isozyme being most abundant (Houslay and Adams, 2003). PDE4 isozymes can occupy discrete spatial subcellular compartments to regulate cAMP-PKA-calcium signaling within specific microdomains (Baillie, 2009). Our previous immunoelectron microscopy (immunoEM) studies of primate layer III dlPFC indicate that PDE4A is concentrated in spines, where it is localized near the spine apparatus, the extension of the calcium-storing smooth endoplasmic reticulum (SER), positioned to regulate cAMP drive on calcium release in close proximity to excitatory synapses (Paspalas et al., 2013). In contrast to the subcellular location of PDE4A on the spine apparatus, PDE4B is frequently localized within the postsynaptic density (PSD) of excitatory synapses, and within dendritic shafts in association with mitochondria (Paspalas et al., 2013). However, the subcellular localization of PDE4D in layer III dlPFC is not known. Understanding PDE4D’s role in the primate dlPFC is particularly important, as PDE4D is enriched in human dlPFC (Carlyle et al., 2017), declines with age in human PFC (Lu et al., 2004), and is a current focus of therapeutics (Baillie et al., 2019). The current study interrogated the subcellular localization of PDE4D in young rhesus macaque dlPFC layer III, the circuits which underlie higher cognition and are a key locus of pathology in schizophrenia and Alzheimer’s disease. We also used laser-capture dissection of neurons in human layer III dlPFC to examine the expression of PDE4D in pyramidal cells vs. parvalbumin (PV) GABAergic interneurons. Our results revealed predominant PDE4D expression in dlPFC pyramidal cells, concentrated in postsynaptic compartments in dendrites on microtubules and the SER, and in dendritic spines near the SER spine apparatus, positioned to regulate cAMP drive on internal calcium signaling.

## Materials and Methods

### Cell-Type Specific Microarray Analysis of PDE4D in Human dlPFC

#### Human Subjects

Brain specimens from seven human subjects with no known neurological or psychiatric disorders were obtained during routine autopsies conducted at the Alleghany County Office of the Medical Examiner (Pittsburgh, PA, United States) after consent was obtained from the next-of-kin. All details relating to sex, age, ethnicity, post-mortem interval, RNA integrity number, brain pH, and tissue storage time for these subjects are available in previously published papers (Arion et al., 2015; Enwright Iii et al., 2018). All procedures were approved by the University of Pittsburgh Committee for the Oversight of Research and Clinical Training Involving Decedents and the University of Pittsburgh Institutional Review Board for Biomedical Research.

#### Laser Microdissection (LMD)

The right hemisphere of each brain was blocked coronally, immediately frozen and stored at −80°C. Tissue sections (12 μm) containing dlPFC area 9 were cut on a cryostat, mounted on glass polyethylene napthalate membrane slides (Leica Microsystems, Bannockburn, IL, United States). For pyramidal cell microdissections, slides were stained with thionin for Nissl substance (Arion et al., 2015) and pyramidal neuron (*n* = 100 cells per sample) cell bodies with a characteristic triangular shape and prominent apical dendrite were identified and dissected from dlPFC layer III using the Leica microdissection system (LMD 6500). For dlPFC layer III parvalbumin-positive GABA interneuron (*n* = 150 cells per sample) dissection, sections were immunolabeled using antibody raised against aggrecan (Enwright Iii et al., 2018), a component of perineuronal nets (PNNs). PNNs are a condensed form of the extracellular matrix around most PV interneurons (Hartig et al., 1992), involved in the closure of developmental critical periods, regulation of synaptic plasticity, and oxidative stress (Cabungcal et al., 2013). Specifically, aggrecan is highly enriched in mature PNNs (Bitanihirwe and Woo, 2014). For each subject, two replicates were processed independently, and replicate samples were averaged for data analysis.

#### Microarray Profiling for Cell-Type Specific Transcript Enrichment

For each sample, RNA was extracted using the QIAGEN Micro RNeasy kit Plus (QIAGEN, Valencia, CA, United States). The Ovation Pico WTA System (San Carlos, CA, United States) was used for synthesis and amplification of cDNA and samples were profiled using the Affymetrix GeneChipU219. Data from all 14 samples were normalized together.

#### Statistical Analyses

Following normalization, average log2 expression values were determined for each probe for a cell type. *P*-values from paired *t*-tests were corrected using the Benjamini-Hochberg procedure, and differential expression determined using a false discovery rate of *q* < 0.05.

### Electron Microscopy of PDE4D in Rhesus Macaque dlPFC Layer III

#### Animals and Tissue Processing

Three female young adult (7, 10, and 11 years) rhesus monkeys (*Macaca mulatta*) in this study were maintained and euthanized in accordance with the guidelines of Yale University Institutional Animal Care and Use Committee and National Institutes of Health “Guidelines for the Care and Use of Experimental Animals.” As described previously (Paspalas and Goldman-Rakic, 2004; Paspalas et al., 2013), the primates were deeply anesthetized prior to transcardial perfusion of artificial cerebrospinal fluid, followed by 4% paraformaldehyde, 0.05% glutaraldehyde, and 0.18% picric acid in 100 mM phosphate buffer. Following perfusion, a craniotomy was performed, and the entire brain was removed and dissected, including a frontal block containing the primary region of interest surrounding the principal sulcus. The brains were sectioned coronally at 60 μm on a vibratome (Leica, Norcross, GA, United States) across the entire rostrocaudal extent of the dorsolateral prefrontal cortex (dlPFC; Walker’s area 46). The sections were cryoprotected through increasing concentrations of sucrose solution (10%, 20%, and 30% each for 2 h, then 30% overnight), cooled rapidly using liquid nitrogen and stored at −80°C. Sections of dlPFC were processed for immunocytochemistry. In order to enable penetration of immunoreagents, all sections went through three freeze-thaw cycles in liquid nitrogen. Non-specific reactivity was suppressed with 10% normal goat serum (NGS) and 2% bovine serum albumin (BSA), and antibody penetration was enhanced with 0.3% Triton X-100 in 50 mM Tris-buffered saline (TBS).

#### Histology and Immunoreagents

We used a previously well-characterized affinity isolated polyclonal primary antibody raised in rabbit against amino acids 156–205 of the PDE4D protein (SAB4502128; Millipore Sigma Aldrich, Burlington, MA, United States) that recognizes human and rodent PDE4D based on sequence homology. The antibody is highly specific and detects endogenous levels of total PDE4D protein at a band migrating at ∼91 kDa. The antibody is suited for a range of applications, including immunohistochemistry, immunoblotting and ELISA as per manufacturer’s recommendations. The specificity and selectivity of the PDE4D antibody has been previously characterized using immunohistochemistry in myocytes to identify a role of PDE4D-PRKAR1α in cardiac contractility (Bedada et al., 2016). The primary antibody was used at 1:200 dilution and was complexed with rabbit-specific goat secondary antibodies. Normal sera and IgG-free BSA were purchased from Jackson ImmunoResearch (West Grove, PA, United States). All chemicals and supplies for electron microscopy were purchased from Sigma Aldrich (St. Louis, MO, United States) and Electron Microscopy Sciences (Hatfield, PA, United States), respectively.

#### Single Pre-embedding Peroxidase Immunocytochemistry

As described previously (Paspalas et al., 2013), the sections were incubated for 72 h at 4°C with primary antibodies in TBS, and transferred for 2 h at room temperature to species-specific biotinylated Fab’ or F(ab’)_2_ fragments in TBS. In order to reveal immunoperoxidase labeling, sections were incubated with the avidin-biotin peroxidase complex (ABC) (1:300; Vector Laboratories, Burlingame, CA, United States) and then visualized in 0.025% 3,3-diaminobenzidine tetrahydrochloride (DAB; Sigma Aldrich, St. Louis, MO, United States) as a chromogen in 100 mM PB with the addition of 0.005% hydrogen peroxide for 10 min. After the DAB reaction, sections were exposed to osmification (concentration 1%), and dehydration through a series of increasing ethanol concentrations (70–100%), infiltrated with propylene oxide. Tissue blocks were counterstained with 1% uranyl acetate in 70% ethanol. Standard epoxy resin embedding followed typical immunoEM procedures followed by polymerization at 60°C for 48 h. Omission of primary antibodies or substitution with non-immune serum resulted in complete lack of immunoperoxidase labeling. Similarly, labeling was nullified when blocking the biotinylated probes with avidin/biotin. Furthermore, we observed a lack of precipitate when control sections were treated with diaminobenzidine.

#### Electron Microscopy and Data Analysis

All sections were processed as previously described (Paspalas et al., 2013). Briefly, blocks containing dlPFC layer III were sampled and mounted onto resin blocks. Our immunoEM analyses focused on dlPFC layer III where the neuropil primarily includes basilar dendrites and proximal apical dendrites from layer III pyramidal cells, but also includes a smaller proportion of distal apical dendrites from pyramidal cells located in layer V. The specimens were cut into 50 nm sections using an ultramicrotome (Leica, Norcross, GA, United States), mounted on individual slot grids and analyzed under a JEM1010 (JEOL, Tokyo, Japan) transmission electron microscope at 80 kV. Individual grids were counterstained with 1% lead citrate. Several plastic blocks of each brain were examined using the 4th to 12th surface-most sections of each block (i.e., 200–600 nm), in order to sample the superficial component of sections, avoiding penetration artifacts. Structures were digitally captured at x25,000-x100,000 magnification of Multiscan 792 camera (Gatan Inc., Pleasanton, CA, United States) and individual panels were adjusted for brightness and contrast using Adobe Photoshop and Illustrator CC.2017.01 image editing software (Adobe Systems Inc., San Jose, CA, United States). Quantitative assessments were performed on a series of low magnification 2D micrographs of supragranular dlPFC layer III, each covering a field of 30 μm^2^ captured from the thin sections; 100 fields/block, 4 blocks/brain for the total of three brains. Approximately, 1200 micrographs of selected areas of neuropil with immunopositive profiles were used for analyses. A total of 2770 PDE4D immunopositive profiles, including dendritic shafts, dendritic spines, axons and astroglia, across three animals were used for quantitative analyses (Table 1). The total number of PDE4D immunopositive profiles was pooled across all blocks for the three young animals. The frequency of PDE4D-positive elements was highly consistent across all three animals, reflected in the small variance for each subcompartment (Table 1). For profile identification, we adopted the criteria summarized by Alan Peters (Peters et al., 1991).

**TABLE 1:** Quantitative assessment of PDE4D immunoreactivity in dlPFC layer III neuropil. Prevalence of PDE4D in cellular compartments.

Subcellular compartment	# of immunopositive profiles	% of immunopositive profiles (Mean ± SEM)
Glia	665	24.0% ± 1.67
Dendrite	1095	39.6% ± 0.82
Spine	527	19.0% ± 0.96
Axon	164	5.9% ± 2.07
N.D.	319	11.5% ± 1.65
**Total**	**2770**	**100%**

*N.D. – Not determined.*

#### Statistical Analysis

To assess the effect of subcompartment on PDE4D immunoEM protein levels, we conducted a repeated-measures analysis of variance (1-ANOVA-R) using the Greenhouse–Geisser correction. We also determined the mean and standard error of mean (SEM) of the protein levels for each subcompartment. Tukey’s *post hoc* test was used for comparisons between cellular subcompartments with α = 0.05 using GraphPad Prism 8.0.

## Results

### Microarray Analyses of Human dlPFC

**FIGURE 1:**
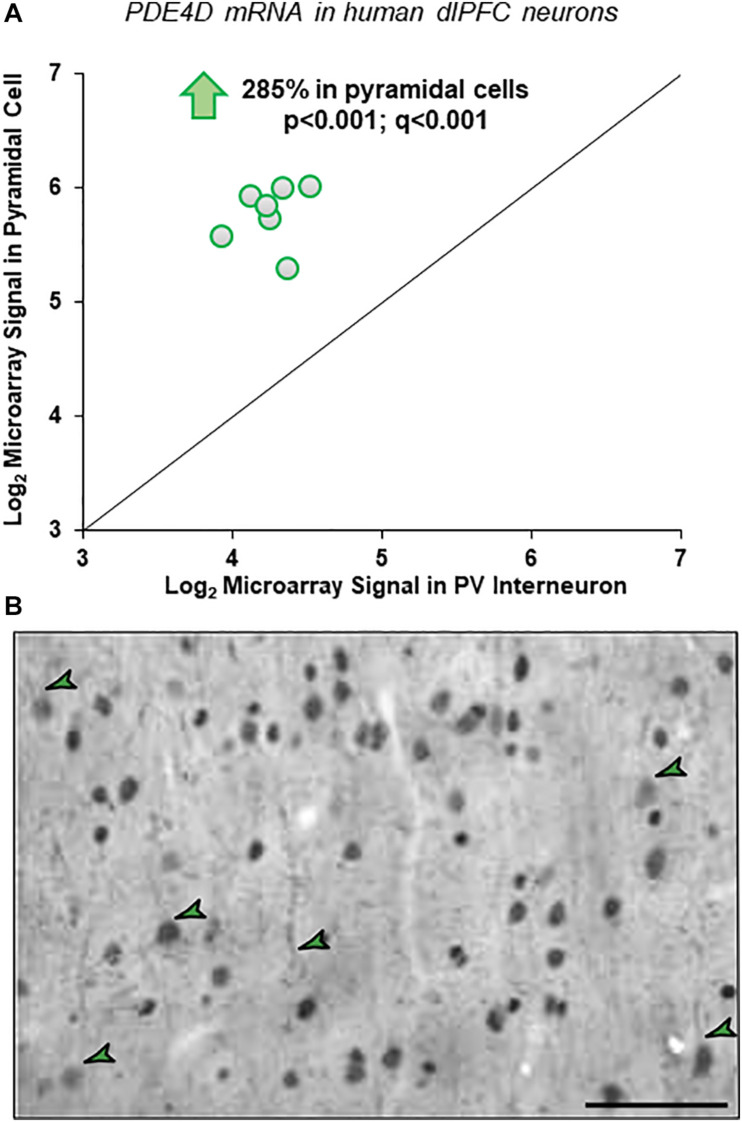
Cell-type specific PDE4D expression in human and macaque dlPFC layer III. **(A)** Human microarray data showing greater PDE4D expression in pyramidal cells vs. PV GABAergic interneurons in human dlPFC layer III. Log_2_-transformed microarray signal of PDE4D mRNA in dlPFC layer III pyramidal and PV cells within human subjects (*N* = 7). The mean log_2_ microarray PDE4D mRNA expression averaged across probes within each subject in pyramidal cells (5.77 ± 0.54) was significantly ∼3-fold higher (*p* < 0.001; *q* < 0.001) compared to PV interneurons (4.26 ± 0.69) in human dlPFC layer III, indicating significantly greater expression of PDE4D mRNA in pyramidal cells from the same subjects. **(B)** Immunohistochemistry for PDE4D immunoreactivity in young macaque dlPFC layer III. The labeling pattern for PDE4D in pyramidal cells is characterized by cytoplasmic staining of the cell body and nuclei, and delicate immunoreactivity along proximal apical dendrites and basal dendrites. PDE4D labeling was also observed in deeper cortical layers. The green arrowheads indicate a few examples where one can observe pyramidal cells with labeled dendrites. Scale bars, 50 μm.

Gene expression data from microarray analyses of layer III pyramidal cells and PV interneurons from human dlPFC demonstrated a 2.85-fold enrichment for PDE4D mRNA in pyramidal cells (Figure 1A). These findings were highly consistent across subjects and across multiple probes, with four of five probes revealing statistically significant enrichment in pyramidal cells vs. PV interneurons across all subjects (*p* < 0.001; *q* < 0.001).

### ImmunoEM in Rhesus Macaque dlPFC

#### Quantitative Mapping of PDE4D Protein in dlPFC Layer III Neuropil

**FIGURE 2:**
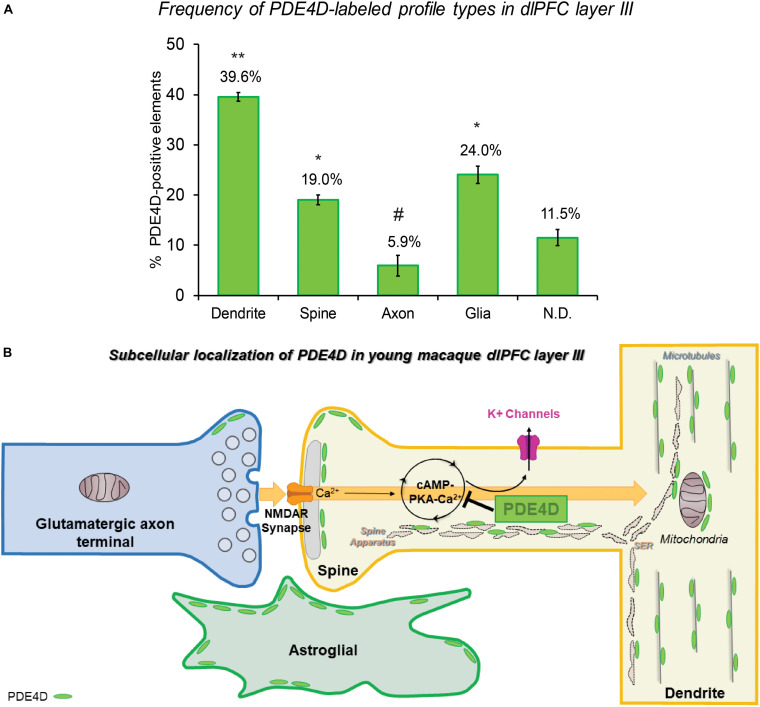
Quantitative analysis of PDE4D localization in macaque dlPFC layer III circuits. **(A)** The prevalence of PDE4D in various cellular subcompartments in layer III of the dlPFC neuropil, expressed as percentage of PDE4D profile (e.g., dendrite) per total PDE4D profiles. PDE4D is primarily expressed in postsynaptic subcompartments in dlPFC layer III microcircuits, with foremost expression in dendritic shafts, and significant expression within dendritic spines and astroglia. For additional details regarding quantitative assessment and profile identification see “Materials and Methods” section. Not determined (N.D) are profiles that could not be unequivocally categorized. ** significantly greater than all other locations; * significantly different from dendrites or axons; # significantly less than all other locations; *p* < 0.05; see text for details. **(B)** Summary schematic of PDE4D expression patterns in dlPFC layer III microcircuits. Neuronal PDE4D is primarily in postsynaptic locations targeted to the most prevalent long, thin spine subtype, positioned to modulate cAMP-PKA-calcium opening of K^+^ channels that are particularly susceptible in schizophrenia and Alzheimer’s disease. The most abundant expression of PDE4D occurs within pyramidal neuron dendritic shafts in association with microtubules, mitochondria and likely the smooth endoplasmic reticulum (SER). PDE4D is localized in astroglia in primate dlPFC layer III, targeted to glial leaflets ensheathing synapses.

**FIGURE 3:**
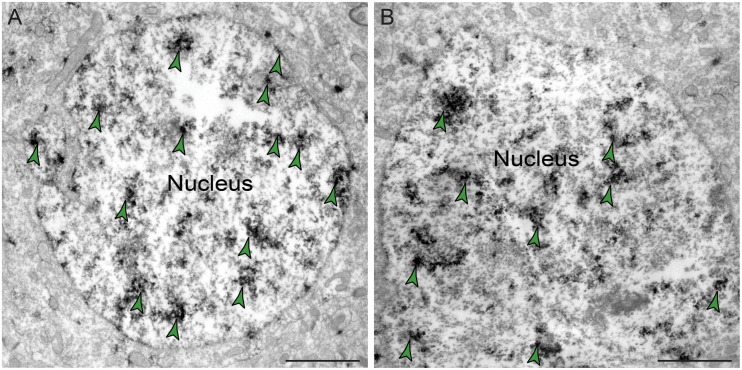
Expression of PDE4D protein within nuclei in dlPFC layer III. **(A,B)** PDE4D protein is visualized within nucleoplasm of round neuronal nuclei in dlPFC layer III. Color-coded arrowheads (green) point to PDE4D immunoreactivity. Scale bars, 2 μm.

The ultrastructural location of PDE4D in layer III neuropil of the rhesus monkey dlPFC was determined using immunoperoxidase labeling. We analyzed a total of 2770 PDE4D-immunopositive profiles, revealing predominantly postsynaptic localization within dendritic shafts and dendritic spines, but only sparse labeling within axons, both synaptic terminals and intervaricose segments (Figure 2A and Table 1). There was also extensive labeling within astroglial cells (Figure 2A and Table 1). Repeated-measures ANOVA with the Greenhouse–Geisser correction revealed a main effect of subcompartment [*F*_(1.323,2.647)_ = 377.6; *P* = 0.0006]. Tukey’s *post hoc* test showed that immunoperoxidase labeling was significantly higher in dendrites (dendrite vs. axons, *P* = 0.0019), spines (spines vs. axons, *P* = 0.0089), and glia (glia vs. axons, *P* = 0.0178) compared to axons. Furthermore, Tukey’s *post hoc* tests revealed that the dendritic subcompartment was significantly higher compared to spines (dendrite vs. spine, *P* < 0.0001) and glia (dendrite vs. glia, *P* = 0.0045). Note that cell nuclei were also labeled (Figure 3), but not counted, as this study focused on localization within neuropil.

Given the prominent postsynaptic labeling, we examined the frequency of PDE4D-labeled spines and dendritic shafts that received Type I (asymmetric, presumed excitatory) vs. Type II (symmetric, presumed inhibitory or neuromodulatory) synapses. The synaptic characteristics can aid in interpretation, e.g., interneurons often receive multiple Type I synapses on their dendritic shafts. We analyzed a total of 1838 dendrites and spines that received at least one synapse, and observed PDE4D labeling within a total of 655 (35.64%) postsynaptic compartments (Table 2). Quantitative assessment of postsynaptic elements receiving a synapse revealed that PDE4D labeling was predominately expressed within dendritic spines receiving a Type I asymmetric glutamatergic-like synapse, with much lower labeling in dendrites receiving Type I or Type II synapses, and scant labeling in spines receiving a Type II synapse (Table 2). The majority of PDE4D-labeled dendritic shafts did not receive either a Type I or Type II synapse (970 profiles, 88.6%; Tables 1,2), consistent with pyramidal cell characteristics. However, among all PDE4D+ postsynaptic dendritic sites, the proportion of dendritic shafts receiving asymmetric synapses was greater than those receiving symmetric synapses. A schematic summary of the subcellular distribution of PDE4D within different cellular subcompartments in dlPFC layer III microcircuits is presented in Figure 2B. The cell-type specific microarray results in human dlPFC corroborate the rhesus macaque immunoEM data, suggesting PDE4D is particularly prominent in pyramidal cell dendritic shafts and spines (see below).

**TABLE 2:** Quantitative assessment of PDE4D-immunopositive dendrites and spines in dlPFC layer III that receive an asymmetric or symmetric synapse.

PDE4D-positive synapse category	Frequency of PDE4D-positive recipient synapses		
Asymmetric synapses	608 (92.82%)	Type I axospinous	527 (80.46%)
		Type I axodendritic	81 (12.37%)
Symmetric synapses	47 (7.18%)	Type II axospinous	3 (0.45%)
		Type II axodendritic	44 (6.72%)
Total		655 (100%)	

#### Predominant Localization of PDE4D Within Dendritic Shafts in dlPFC Layer III

**FIGURE 4:**
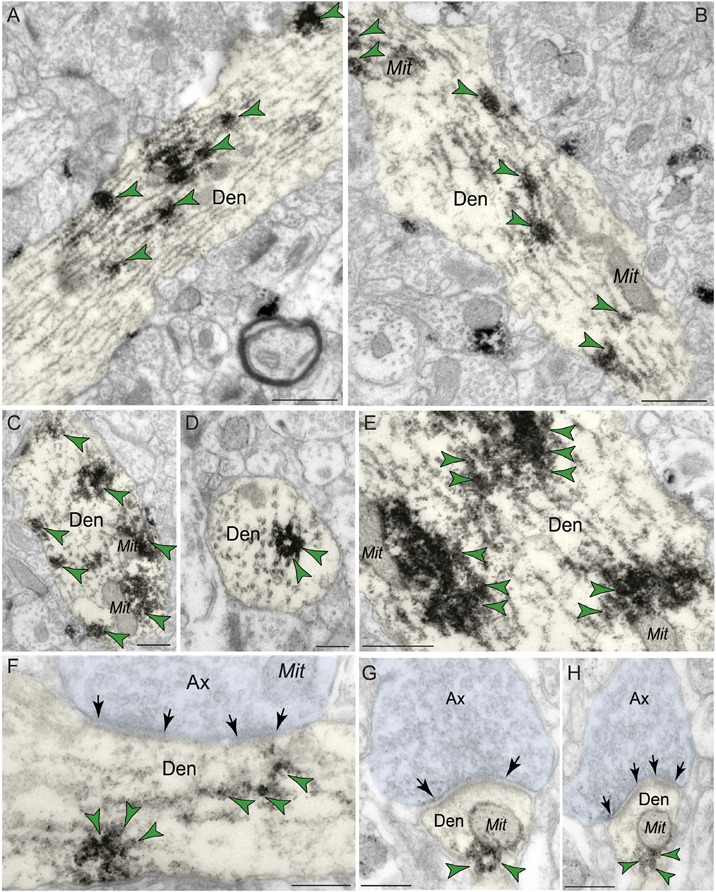
Postsynaptic expression of PDE4D within dendritic shafts in dlPFC layer III. **(A–F)** In young macaque dlPFC layer III, PDE4D was predominantly concentrated in dendritic shafts in postsynaptic locations and was associated with microtubules oriented in parallel bundles and likely SER tubules. In **(F)**, the dendritic shaft receives an axodendritic asymmetric glutamatergic-like Type I synapse. **(G,H)** PDE4D immunolabeling within dendritic shafts was associated with mitochondrial profiles. All dendritic shafts receive axodendritic asymmetric Type I glutamatergic-like synapses. Synapses are between arrows. Color-coded arrowheads (green) point to PDE4D immunoreactivity. Profiles are pseudocolored for clarity. Ax, axon; Den, dendrite; Mit, mitochondria. Scale bars, 200 nm.

Immunoreactivity for PDE4D was most prevalent within dendritic shafts in dlPFC layer III neuropil (Table 1), likely from pyramidal cells located in layer III. PDE4D label was observed in pyramidal cell dendrites with light microscopy (Figure 1B), and ultrastructural analysis confirmed PDE4D localization within the proximal segment of apical dendrites and basilar branches (Figures 4A–C,E). Many of the dendrites had the morphological characteristics of pyramidal cells, given their large diameter and ruffled/spiny membrane (Figures 4A–C,E), consistent with the light microscopy and the transcriptomic data from human layer III dlPFC. Within dendrites, PDE4D labeling was primarily associated with parallel bundles of microtubules within dendritic shafts, which could be seen in both horizontal (Figures 4A–C,E,F) and perpendicular (Figure 4D) planes. Although the dense labeling obscured underlying details, it is likely that PDE4D immunoreactivity also was associated with the SER, given the frequent concentration adjacent to mitochondria (Figures 4B,C,E,G,H), a common focus of SER tubules. PDE4D was often localized within the cytosol of small dendritic shafts receiving axodendritic Type I asymmetric glutamatergic-like synapses (Figures 4G,H) which may be either the distal dendrioles of pyramidal cells and/or interneuronal dendrites. Immunolabeling for PDE4D was observed to a lesser extent in dendritic shafts receiving axodendritic Type II symmetric synapses (Table 2).

#### PDE4D Is Enriched Within Dendritic Spines in dlPFC Layer III

**FIGURE 5:**
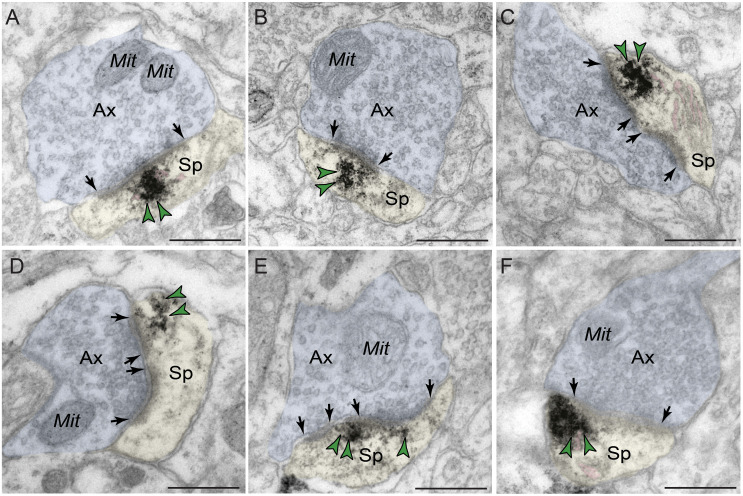
Postsynaptic expression of PDE4D within dendritic spines in dlPFC layer III. PDE4D immunolabeling is prominently expressed in dendritic spines, subjacent to the PSD near the synaptic active zone **(A–C,E)**, and associated with, or in close proximity to the calcium-storing spine apparatus pseudocolored in pink **(A,C,F)**. PDE4D immunolabeling is also observed in perisynaptic or extrasynaptic compartments near the plasma membrane flanking the excitatory synapse **(C,D,F)**. All dendritic spines receive axospinous Type I asymmetric glutamatergic-like synapses. Synapses are between arrows. Color-coded arrowheads (green) point to PDE4D immunoreactivity. Profiles are pseudocolored for clarity. Ax, axon; Mit, mitochondria; Sp, dendritic spine. Scale bars, 200 nm.

Phosphodiesterase 4D labeling was also localized within dendritic spines in macaque dlPFC layer III (Figure 5 and Tables 1,2), putatively arising from pyramidal cells located in layer III. PDE4D was primarily concentrated in mature, likely thin-type dendritic spines receiving Type I glutamatergic-like asymmetric synapses with sparse labeling near Type II axospinous symmetric synapses. The relatively small dendritic spine head is consistent with thin spine subtype, and the appearance of a spine apparatus and/or perforated synapse is consistent with a mature synapse. PDE4D labeling was often observed subjacent to the PSD, exhibiting non-uniform expression along the length of the synaptic active zone (Figures 5A–C,E). PDE4D labeling also was found at perisynaptic and extrasynaptic membranes flanking the glutamatergic-like synapse (Figures 5C,D,F). Importantly, PDE4D labeling was visualized in association with, or near the spine apparatus, the calcium-containing extension of the SER into the spine head, positioned to regulate cAMP-PKA-calcium signaling (Figures 5A,C,F).

#### Extensive Localization of PDE4D Within Astroglia in dlPFC Layer III

**FIGURE 6:**
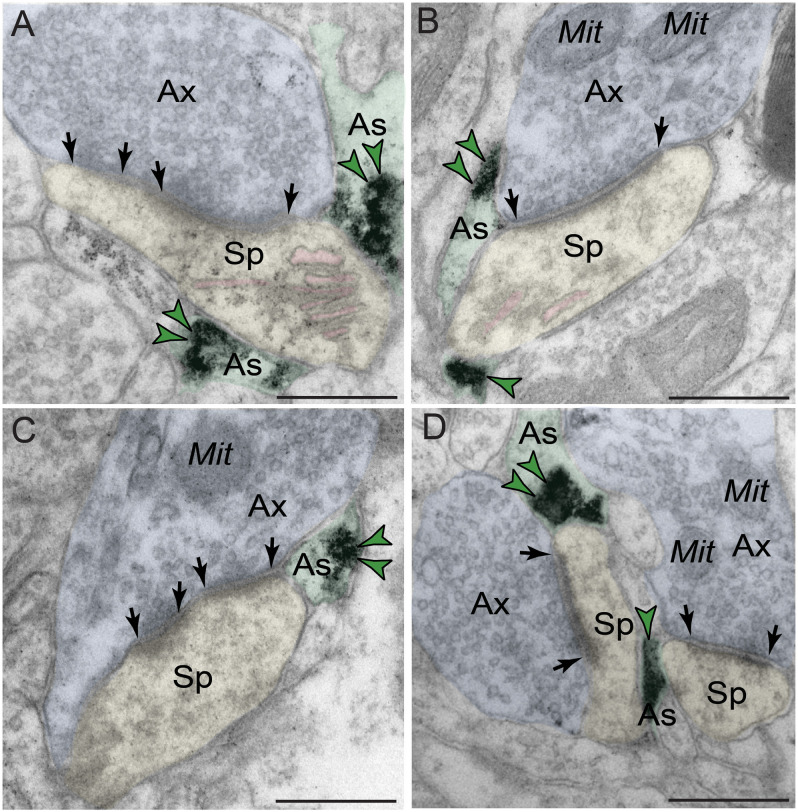
Astroglial expression of PDE4D in dlPFC layer III. **(A–D)** Immunolabeling for PDE4D is visualized within astroglial leaflets ensheathing glutamatergic synapses. PDE4D deposits appear to show a predilection for the astroglial leaflets near the plasma membrane in perisynaptic locations near the excitatory synapse. All dendritic spines receive axospinous Type I asymmetric glutamatergic-like synapses, and the spine apparatus is pseudocolored in pink in the spine head. Synapses are between arrows. Color-coded arrowheads (green) point to PDE4D immunoreactivity. Profiles are pseudocolored for clarity. Ax, axon; Mit, mitochondria; Sp, dendritic spine; As, astroglia. Scale bars, 200 nm.

Phosphodiesterase 4D also was robustly expressed in astroglia (Figure 6 and Table 1). PDE4D labeling was targeted to perisynaptic astroglial leaflets, ensheathing axospinous glutamatergic-like synapses (Figures 6A–D). Immunolabeling for PDE4D within astroglia was not uniformly distributed along the plasmalemma, but was instead concentrated near the synapse.

#### Sparse Labeling of PDE4D in Axon Terminals in dlPFC Layer III

**FIGURE 7:**
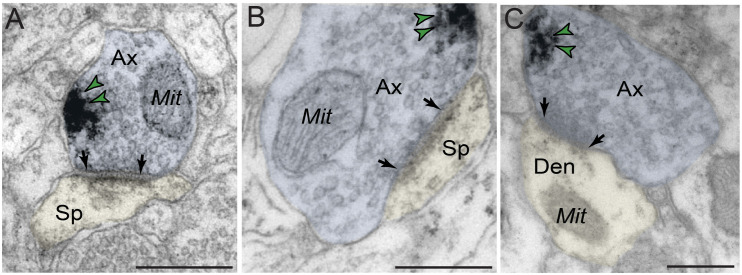
Presynaptic expression of PDE4D in dlPFC layer III. **(A,B)** PDE4D is expressed in glutamatergic-like axons establishing axospinous Type I asymmetric synapses. The labeling is found in perisynaptic and extrasynaptic subcompartments near the axon terminal plasmalemma, and not typically within the synaptic active zone. **(C)** PDE4D is expressed in glutamatergic-like axons establishing axodendritic Type I asymmetric synapses. Synapses are between arrows. Color-coded arrowheads (green) point to PDE4D immunoreactivity. Profiles are pseudocolored for clarity. Ax, axon; Mit, mitochondria; Den, dendrite; Sp, dendritic spine. Scale bars, 200 nm.

In contrast to robust prevalence in postsynaptic neuronal compartments, PDE4D labeling in presynaptic neuronal subcompartments in dlPFC layer III neuropil was infrequent (Figure 7 and Table 1). In the small subset of axon terminals that were immunopositive for PDE4D, the labeling was observed in perisynaptic or extrasynaptic subcompartments bordering the excitatory synapse (Figures 7A–C). PDE4D labeling was visualized in association with the plasma membrane in glutamatergic-like axon terminals forming axospinous Type I asymmetric synapses (Figures 7A,B), and axodendritic Type I asymmetric synapses (Figure 7C).

#### Summary of ImmunoEM Findings

In aggregate, ultrastructural analyses demonstrated that PDE4D exhibits a distinct distribution pattern in dlPFC layer III microcircuits, reflecting specificity in cell-types, subcompartments and interaction with subcellular organelles (Figure 2B). PDE4D was predominantly expressed in postsynaptic compartments, within dendritic shafts in association with microtubules, mitochondria, and likely SER. Moreover, PDE4D protein was highly prevalent in postsynaptic compartments within thin-type dendritic spines, with specific synaptology (e.g., glutamatergic Type I asymmetric synapses). The presence of PDE4D labeling near the calcium-storing SER spine apparatus within dendritic spines in pyramidal cells would be consistent with the role of cAMP-PKA modulation of internal calcium release. Astroglia also exhibited PDE4D immunolabeling, in perisynaptic locations near axospinous Type I asymmetric synapses. Finally, we observed limited presynaptic localization of PDE4D within glutamatergic-like axon terminals.

## Discussion

This study provides the first description of the anatomical localization of PDE4D in the newly evolved microcircuits of primate dlPFC that produce the mental representations underlying working memory. The results indicate that PDE4D is positioned to regulate cAMP signaling at key postsynaptic locations in dendrites and spines, as well as in perisynaptic astrocytes, highlighting a role in the modulation of glutamatergic signaling in layer III dlPFC. Transcriptomic analysis of the same microcircuits in human layer III dlPFC demonstrated that PDE4D is particularly enriched in pyramidal cells, consistent with the electron microscopy data from non-human primates. Altogether, these findings indicate that PDE4D expression in primate layer III dlPFC is positioned to regulate cAMP signaling in the recurrent excitatory circuits that generate working memory.

### Microdomains of cAMP Signaling Within Cells

The current findings bolster the notion that the spatial and temporal dynamics of cAMP signaling are regulated by discretely positioned phosphodiesterases that act as sinks to create simultaneous cAMP gradients in multiple cellular microdomains (Conti et al., 2003; Houslay and Adams, 2003; Baillie, 2009). For example, in mouse embryonic fibroblasts, PDE4A and PDE4B regulate cAMP in discrete subdomains near the plasma membrane, while PDE4D modulates bulk cytosolic cAMP signaling (Conti et al., 2003; Blackman et al., 2011). Microdomains of cAMP signaling have also been seen in layer III of rhesus macaque dlPFC, with PDE4B documented in the PSD and near dendritic mitochondria, while PDE4A is concentrated in spines near the SER spine apparatus (Paspalas et al., 2013; Carlyle et al., 2014). The current study also found microdomains defined by PDE4D within neurons and glia. For example, PDE4D had very focal expression in perisynaptic astrocytic processes immediately next to the synapse. PDE4D expression in this glial microdomain may regulate glutamate uptake from the synapse as has been reported in mouse cortex, where PDE4D regulates cAMP mediated trafficking of the excitatory amino acid transporters (EAAT) that control glutamate levels in the synapse (Hughes et al., 2004; Zhang et al., 2014; Sharma et al., 2015; Zhou et al., 2019).

### Regulation of Postsynaptic Signaling in Dendrites by PDE4D

The most robust labeling for PDE4D was observed within dendritic shafts, particularly in association with microtubules, mitochondria and likely SER tubules. It is likely that many of these PDE4D-expressing dendrites are from pyramidal cells, given their morphological characteristics (e.g., large size, ruffled edges consistent with a spiny membrane), and the light-level immunohistochemistry showing delicate labeling of putative pyramidal cell dendrites. This interpretation would be consistent with the human transcriptomics, demonstrating ∼3-fold greater expression of PDE4D in pyramidal cells than GABAergic PV interneurons in human dlPFC layer III (for human subject cohort see Table 3). Future studies could perform double labeling to identify the PDE4D role in interneurons and cell-type specific transcriptomics in young rhesus macaque dlPFC layer III.

**TABLE 3:** Demographic, postmortem, and clinical characteristics of human subjects used in this study.

Subject	Case	Sex/Race	Age	PMI^*a*^	Storage time^*b*^	RIN	pH	BMI	Cause of death
1	1047	M/W	43	13.8	134.0	9.0	6.6	29.4	ASCVD
2	1086	M/W	51	24.2	128.0	8.1	6.8	25.5	ASCVD
3	1247	F/W	58	22.7	108.7	8.4	6.4	35.9	ASCVD
4	1324	M/W	43	22.3	95.1	7.3	7	30.9	Aortic dissection
5	1391	F/W	51	7.8	84.1	7.1	6.6	28.3	ASCVD
6	1282	F/W	39	24.5	102.9	7.5	6.8	30.6	ASCVD
7	686	F/W	52	22.6	197.5	8.5	7.0	U	ASCVD

*^*a*^PMI, postmortem interval (hours); ^*b*^Storage time (months) at −80°C; ASCVD, arteriosclerotic cardiovascular disease; BMI, Body Mass Index; RIN, RNA Integrity Number; U, unknown.*

The subcellular localization of PDE4D within dendrites suggests multiple potential functions. The dense labeling near microtubules suggests that PDE4D is not simply trafficking on microtubules, but is likely regulating microtubule dynamics and/or trafficking along bundles. To our knowledge, there is little known about cAMP modulation of trafficking along microtubules; the enrichment of PDE4D on these organelles suggests this is an arena that warrants further research. One likely role for PDE4D at this site may be the regulation of cAMP-PKA phosphorylation of tau, a protein that influences microtubule stability and dynamics (Panda et al., 2003; Barbier et al., 2019; Venkatramani and Panda, 2019). As described below, microtubules are a common location for PKA-phosphorylated tau in aging monkey dlPFC (Carlyle et al., 2014).

The definitive localization of PDE4D on the SER in dendrites would require double-labeling with a SER calcium channel, i.e., ryanodine receptors (RyRs) or inositol 1, 4, 5-triphosphate receptors (IP3Rs), but the frequent concentration of PDE4D label on tubules near mitochondria would be consistent with this hypothesis. If this idea is correct, PDE4D would be positioned to regulate cAMP drive on calcium entry into mitochondria, which influences mitochondrial dynamics, energy production, and inflammation. For example, SER tubules play an active role in defining sites of mitochondrial fission and fusion (Westermann, 2010; Friedman et al., 2011; Wu et al., 2017). SER-mitochondria interfaces also play an important role in coordinating calcium transients, inflammasome formation, and autophagosome assembly (Zhou et al., 2011; Lee et al., 2012a; Hamasaki et al., 2013). The current data suggest that PDE4D is positioned to regulate these important cAMP actions, and may help to prevent calcium overload of mitochondria that can initiate inflammatory signaling. Microdomains of cAMP signaling regulated by PDE4D within pyramidal neurons might also regulate calcium waves through the SER in dendritic shafts. These calcium waves are initiated by activation of type 1 metabotropic glutamate receptors (mGluRs), which mobilize calcium release from the SER through RyRs and IP3Rs (Watanabe et al., 2006). Therefore, the spatial compartmentalization of cAMP-calcium by PDE4D within dendritic shafts could dictate the propagation of these calcium waves that invade the soma, and modulate backpropagating action potentials (Houslay and Adams, 2003; Watanabe et al., 2006).

### PDE4D Positioned to Regulate cAMP-PKA-Calcium-K^+^ Channel Signaling in Spines

Phosphodiesterase 4D was also prominently localized within layer III dendritic spines, concentrated on the SER spine apparatus, often subjacent or next to the PSD. PDE4B is also localized within or next to the PSD (Paspalas et al., 2013), a site where cAMP-PKA signaling may modulate trafficking of receptors and ion channels in and out of the synaptic membrane (Zheng and Keifer, 2009; Song et al., 2013). The calcium-storing spine apparatus is also a common focus of cAMP signaling, and is a major focus of PDE4A expression (Paspalas et al., 2013; Carlyle et al., 2014). PDE4D was also localized on the spine apparatus, where it may have dynamic influence on the strength of synaptic connectivity through feedforward, calcium-cAMP-PKA opening of nearby potassium (K^+^) channels (Arnsten et al., 2012; Arnsten, 2015). Thus, in contrast to cAMP signaling in classic circuits, feedforward cAMP-PKA-calcium signaling in dlPFC circuits reduces working memory-related neuronal firing through the opening of K^+^ channels, as seen with cAMP analogs or PDE4 inhibitors (Wang et al., 2007; Carlyle et al., 2014). These data caution that the development of PDE4D inhibitors as potential therapeutics for cognitive disorders may be problematic for treating PFC cognitive deficits, and that PDE4D may be needed to maintain strong spine connections in layer III dlPFC.

### Clinical Relevance to the Neuropathology of Schizophrenia and Alzheimer’s Disease

The dlPFC layer III pyramidal cell circuits are a primary locus of pathology in a number of higher cognitive disorders, including schizophrenia and age-related cognitive disorders such as sporadic Alzheimer’s disease. Human neuroimaging studies have revealed hypofrontality of dlPFC in patients with schizophrenia during a working memory task that strongly correlates with symptoms of thought disorder (Perlstein et al., 2001). Postmortem studies of the dlPFC from subjects with schizophrenia have corroborated these findings revealing a decrement in the density of dendritic spines and dendritic arbors in dlPFC deep layer III (Garey et al., 1998; Glantz and Lewis, 2000), and associated with mitochondrial dysfunction and a hypometabolic phenotype (Arion et al., 2015; Hoftman et al., 2017). Directly relevant to the current study, single-nucleotide polymorphisms (SNPs) in *PDE4D* that decrease mRNA expression are associated with increased risk of schizophrenia and cognitive impairment in mental illness (Tomppo et al., 2009; Sinha et al., 2019). *PDE4D* has been etiologically implicated in the pathogenesis of other neuropsychiatric diseases associated with PFC dysfunction, with genome-wide association studies identifying point mutations in PDE4D with intellectual disability, major depression and anxiety disorders (Shifman et al., 2008; Lee et al., 2012b; Lindstrand et al., 2014). In addition, PDE4D interacts with another risk gene for mental illness, Disrupted in Schizophrenia 1 (DISC1) (Murdoch et al., 2007; Chubb et al., 2008), which anchors PDE4s to the SER where it can regulate cAMP drive on internal calcium release (Kirkpatrick et al., 2006; Paspalas et al., 2013).

The highly recurrent excitatory circuits in dlPFC layer III are particularly vulnerable with advancing age with selective loss of long-thin dendritic spines in human and macaque (Morrison and Baxter, 2012; Peters and Kemper, 2012) and gray matter atrophy in Alzheimer’s disease (AD) (Bussiere et al., 2003). Our data have indicated that loss of PDE4s with advancing age can dysregulate cAMP signaling and contribute to loss of neuronal firing, impaired working memory and tau phosphorylation (Ramos et al., 2003; Wang et al., 2011; Carlyle et al., 2014). For example, PDE4A is lost from dendritic spines with advancing age, and is associated with increased PKA-mediated phosphorylation of tau at serine-214 (pS214-tau) (Carlyle et al., 2014). In aged macaque dlPFC layer III, we observe pS214-tau accumulating along microtubules and on the spine apparatus, the same subcellular organelles where we find PDE4D in young macaque dlPFC layer III. pS214-tau is particularly important because it causes tau to detach from microtubules and aggregate (Jicha et al., 1999), priming tau for hyperphosphorylation by the kinase, GSK3β (Liu et al., 2006), eventually fibrillating as paired helical filaments in neurofibrillary tangles (Braak and Braak, 1991). The expression, activity and proper localization of PDE4 isoforms may be compromised with advancing age by activated GSK3β (Zhu et al., 2010), and by inflammatory MAPK-activated protein kinase 2 (MK2) signaling, which attenuates activity and reduces PDE4 interaction with its anchoring proteins (Mackenzie et al., 2011; Houslay et al., 2017). As PDE4D inhibitors are currently under development for the treatment of sporadic AD, the current results caution that these agents may exacerbate rather than slow the degenerative process in aging dlPFC circuits.

### Conclusion

In summary, PDE4D is strategically positioned to regulate cAMP signaling in dendrites, spines and astrocytes, with particular focus on the glutamatergic microcircuits that subserve working memory. Given the interest in PDE4D as a therapeutic target for cognitive disorders, better understanding of its precise anatomical localization in primate dlPFC should help to guide more informed strategies for treating higher cognitive deficits.

## Data Availability Statement

The original contributions presented in the study are publicly available. This data can be found here: GEO accession numbers GSE93577 and GSE87610.

## Ethics Statement

The studies involving human participants were reviewed and approved by the University of Pittsburgh Committee for the Oversight of Research and Clinical Training Involving Decedents University of Pittsburgh Institutional Review Board for Biomedical Research. The patients/participants provided their written informed consent to participate in this study. The animal study was reviewed and approved by the Yale University Institutional Animal Care and Use Committee.

## Author Contributions

DD designed, performed, and analyzed immunoEM and immunohistochemistry experiments in rhesus macaques. JE and DA collected and analyzed cell-type specific laser-capture microdissection and microarray experiments in human subjects. CP and YM contributed to the experimental design, provided technical expertise, and revised the manuscript. AA and DL designed the experiments, supervised the study, and critically revised the manuscript. All authors read and approved the final manuscript.

## Conflict of Interest

The authors declare that the research was conducted in the absence of any commercial or financial relationships that could be construed as a potential conflict of interest.
